# Protein Expression of DNA Damage Repair Proteins Dictates Response to Topoisomerase and PARP Inhibitors in Triple-Negative Breast Cancer

**DOI:** 10.1371/journal.pone.0119614

**Published:** 2015-03-16

**Authors:** Julie L. Boerner, Nicole Nechiporchik, Kelly L. Mueller, Lisa Polin, Lance Heilbrun, Scott A. Boerner, Gina L. Zoratti, Karri Stark, Patricia M. LoRusso, Angelika Burger

**Affiliations:** 1 Department of Oncology, Wayne State University, Barbara Ann Karmanos Cancer Institute, Detroit, Michigan, United States of America; 2 Department of Oncology, Yale University, New Haven, Connecticut, United States of America; University Medical Center Hamburg-Eppendorf, GERMANY

## Abstract

Patients with metastatic triple-negative breast cancer (TNBC) have a poor prognosis. New approaches for the treatment of TNBC are needed to improve patient survival. The concept of synthetic lethality, brought about by inactivating complementary DNA repair pathways, has been proposed as a promising therapeutic option for these tumors. The TNBC tumor type has been associated with *BRCA* mutations, and inhibitors of Poly (ADP-ribose) polymerase (PARP), a family of proteins that facilitates DNA repair, have been shown to effectively kill *BRCA* defective tumors by preventing cells from repairing DNA damage, leading to a loss of cell viability and clonogenic survival. Here we present preclinical efficacy results of combining the PARP inhibitor, ABT-888, with CPT-11, a topoisomerase I inhibitor. CPT-11 binds to topoisomerase I at the replication fork, creating a bulky adduct that is recognized as damaged DNA. When DNA damage was stimulated with CPT-11, protein expression of the nucleotide excision repair enzyme ERCC1 inversely correlated with cell viability, but not clonogenic survival. However, 4 out of the 6 TNBC cells were synergistically responsive by cell viability and 5 out of the 6 TNBC cells were synergistically responsive by clonogenic survival to the combination of ABT-888 and CPT-11. *In vivo*, the *BRCA* mutant cell line MX-1 treated with CPT-11 alone demonstrated significant decreased tumor growth; this decrease was enhanced further with the addition of ABT-888. Decrease in tumor growth correlated with an increase in double strand DNA breaks as measured by γ-H2AX phosphorylation. In summary, inhibiting two arms of the DNA repair pathway simultaneously in TNBC cell lines, independent of *BRCA* mutation status, resulted in un-repairable DNA damage and subsequent cell death.

## Introduction

Triple-negative breast cancers (TNBCs) fall into the basal breast cancer subtype and lack estrogen receptor (ER), progesterone receptor (PR), and HER2 expression and activation [1]. While estrogen and HER2 targeting molecules have improved survival rates for luminal and HER2 breast cancer subtypes, significant advancement in targeted therapy for TNBC has yet to be demonstrated [2]. Features of TNBC that may direct the development of targeted therapeutics for this disease include epidermal growth factor receptor (EGFR) overexpression, enhanced angiogenesis, and *BRCA* mutations [3].

The *BRCA* family of genes are tumor suppressors. When mutated, these genes are associated with familial breast and ovarian cancer. The BRCA protein has been shown to be important in DNA repair, regulation of transcription, and ubiquitination [4]. Recently, it has been predicted that sporadic breast cancers may also contain alterations in *BRCA* genes [5]. In fact, in an evaluation of 360 sporadic breast cancers, 80 tumors had *BRCA* mutations [5]. Further, 54% of these 80 tumors were TNBCs, suggesting a high prevalence of sporadic *BRCA* mutations in TNBC [5]. Changes in clinical guidelines now suggest that women with TNBC under the age of 60 be screened for *BRCA* mutations [6].

The BRCA family of proteins have been shown to have many cellular functions, including the regulation of DNA damage repair by homologous recombination [7]. Specifically, BRCA proteins recognize bulky adducts and cross-linked strands of DNA and work within a large complex of proteins to remove damaged DNA and replace the proper nucleotides through homologous recombination with complementary strands of DNA [7]. It is through this mechanism of DNA damage repair that BRCA proteins are thought to work as tumor suppressors. When DNA damage occurs in the absence of BRCA protein expression, DNA containing replication errors may result in genetic mutations not compatible with cell viability [8].

Poly(ADP-ribose) polymerase (PARP) is a DNA binding protein that scans DNA strands for damage [9]. Once damage has been recognized, PARP binds to the DNA and recruits x-ray repair complementation group 1(XRCC1) and tyrosol DNA phosphodiesterase 1 (TDP1) to remove the damaged region of DNA, enabling repair proteins to fill-in the missing nucleotides [9]. Small molecule PARP inhibitors have been identified and used to abrogate DNA damage repair using both *in vitro* and *in vivo* model systems [10]. However, cells contain alternative mechanisms for repairing damage in the absence of PARP activity, including nucleotide excision repair and homologous recombination [11]. In that regard, cells containing mutations in proteins involved in nucleotide excision repair or homologous recombination have an increased sensitivity to PARP inhibitors via a process referred to as *synthetic lethality* [8]. *BRCA* mutated cells exhibit enhanced synthetic lethality with PARP inhibitors and have shown promise in the clinical treatment of *BRCA* mutated tumors [12].

Here we have assessed the efficacy of combining the PARP inhibitor ABT-888 with the DNA damaging topoisomerase I inhibitor, CPT-11 [13]. CPT-11 damages DNA by binding to topoisomerase I and preventing the unwinding of DNA required for DNA replication [14]. This results in a stalled replication fork that can be repaired by PARP. Here we show that adding ABT-888 to CPT-11 decreased cell viability and increased DNA double-strand breaks in TNBC cell lines *in vitro* and *in vivo*. In addition, we identified loss of ERCC1 protein expression in the context of PARP1 protein expression as a marker of resistance to this drug combination. In summary, these data provide supportive preclinical data that inhibiting topoisomerase I and PARP1 in combination, as was demonstrated with the combination of ABT-888 and CPT-11, may result in synergistic decreases in tumor regression for women with TNBC.

## Materials and Methods

### Reagents

ABT-888 was supplied by Abbott Laboratories (Abbott Park, IL) under the Clinical Trials Agreement with the Division of Cancer Treatment and Development at the National Cancer Institute (Bethesda, MD). ABT-888 was dissolved in dimethylsulphoxide (DMSO) to make a stock concentration of 10mM and stored at -20°C. Irinotecan/CPT-11 was purchased commercially (Hospira Inc, Lake Forest, IL). It was also dissolved in DMSO to a concentration of 10 mM and stored at -20°C. All other reagents were purchased from Sigma (St. Louis, MO) unless otherwise noted.

### Cell lines and culture conditions

SUM149, SUM159, and SUM1315 were provided by Dr. Stephen Ethier [15]. HCC1937 and MDA-MB-231 were purchased from ATCC (Manassas, VA). MX-1 was obtained from the NCI Cell Line Repository, Rockville, MD. Medias were purchased from Invitrogen (Grand Island, NY). SUM149 and SUM159 cells were cultured in Ham’s F-12 media supplemented with 5% FBS, 1 μg/ml hydrocortisone, and 5 μg/ml insulin. SUM 1315 cells were grown in Ham’s F-12 media, supplemented with 5% FBS, 10 ng/ml EGF, and 5 μg/ml insulin. HCC1937 and MX-1 cells were grown in RPMI-1640 media with 2 mM L-glutamine adjusted to contain 1.5 g/L sodium bicarbonate, 4.5 g/L glucose, 10mM HEPES, 1mM sodium pyruvate, and 10% FBS. MDA-MB-231 cells were grown in DMEM media containing 10% FBS. The cells were passaged routinely and grown under standard conditions. Logarithmically growing cells were used for all experiments.

### Cell viability assays

Exponentially growing cells were seeded in 96-well plates (MX-1: 5,000 per well, all others: 2,000 per well) and the single agent drugs (ABT-888 or CPT-11) were added in concentrations ranging from 0.01 nM to 100 μM the following day. When the drugs were used in combination, CPT-11 was added to media with a constant ABT-888 concentration of 500nM. Cell proliferation was determined 5 days after continuous exposure to drug by addition of 3-(4,5-dimethylthiazol-2-yl)-2,5-diphenyltetrazoliumbromide (MTT; Promega: Madison, WI). The conversion of MTT to purple formazan by viable cells was measured using the Synergy2 microplate reader and GEN5 microplate data collection and analysis software (Biotex Instruments, Inc. VT). Growth curves were generated as percent of control and 50 and 100% growth inhibitory concentrations determined.

### Clonogenic survival assays

Exponentially growing cells were seeded in 6-well plates and treated with fixed concentrations of ABT-888, CPT-11, or the combination every other day for 1 week. Cells were then trypsinized and replated at low densities in triplicate in 6-well plates and cultured under normal growth conditions for two weeks. Colonies were stained with crystal violet and counted using GelCount colony counter and associated software.

### IC_50_, LD_50_ and synergy calculations

IC_50_ and LD_50_ values were calculated using Calcusyn software (Biosoft, Cambridge, UK). Data from the cell viability or clonogenic assays were converted to surviving fraction by dividing the absorbance values for each dose of drug by the average of the vehicle treated cells. To obtain the fraction of cells affected (Fa) by the treatment, the surviving fraction values were subtracted from 1. The Fa numbers were entered into the software program and IC_50_ or LD_50_ values were generated based on construction of a sigmoidal dose response curve. From these data, synergy calculations were performed using this software as described by Chou and Talalay [16]. Combinatorial index (CI) values were calculated using the viability data from at least three experiments performed in triplicate.

### Immunoblotting

Lysates were prepared from the indicated cells using a CHAPs based lysis buffer (10 mM CHAPs, 50 mM Tris, pH 8.0, 150 mM NaCl, and 2 mM EDTA with 10 μM NaOVa and 1X protease inhibitor cocktail (Calbiochem: Billerica, MA)). Protein was quantified and equal amounts of protein from each cell lysate were separated by SDS-PAGE and transferred to Immobolin P (Millipore: Billerica, MA). Immunoblots were blocked in 5% non-fat dry milk for 1 hr at room temperature (RT) and incubated overnight with primary antibody (anti-PARP, Cell Signaling: Boston, MA, 1:1000; anti-ERCC1, NeoMarkers (ThermoFisher): Waltham, MA, 1:1000). Immunoblots were washed 3X for 10 min, incubated with the appropriate secondary antibody linked to horseradish peroxidase (HRP; Cell Signaling) for 1 hr at RT, washed 3X for 10 min, and developed using ECL (GE Healthcare Biosciences: Piscataway, NJ).

### siRNA knockdown and BrdU incorporation assay

Cells were plated on coverslips in 6-well plates. ERCC1 was knocked down using four non-overlapping siRNA constructs as well as a negative control construct from Dharmacon (Fisher Scientific). (Parallel plates were lysed, lysates were separated by SDS-PAGE, and immunoblotted for ERCC1 to determine knockdown efficiency). For the siRNA transfection, cells were incubated with 7.5 nM of the indicated siRNA (ERCC1 siRNA #1 = CGACGUAAUUCCCGACUAU, ERCC1 siRNA #2 = GGCGGUACCUGGAGACCUA, ERCC1 siRNA #3 = GGAAGAAAUUUGUGAUACC, and ERCC1 siRNA #4 = GCAAUCCCGUACUGAAGUU) with 5 μl Dharmafect for 48 hr. Cells were then placed in serum free media for 18 hours to synchronize and pulsed with BrdU for 4 hrs. Cells were fixed to coverslips with paraformaldehyde and nuclei were pierced with 2N HCl for 2 hrs at 37C. Acid was neutralized with borate washed and BrdU incorporation was detected after blocking in 20% goat serum and incubating with anti-BrdU linked to Alexa-Fluor 624. Coverslips were mounted onto slides with mounting media containing DAPI and BrdU positive nuclei were counted.

### Xenograft tumor growth

**In vivo tumor model establishment and maintenance.** This study was carried out in strict accordance with the recommendations in the Guide for the Care and Use of Laboratory Animals of the National Institutes of Health. Animals were supplied food and water *ad libitum* and were housed in a fully accredited AAALAC animal facility under the care and direction of full-time licensed and board certified staff veterinarians and veterinary technicians. The protocol was approved by the animal use and care committee of Wayne State University (Permit Number: A3310–01). All efforts were made to minimize suffering. Tumors were maintained *in vivo* in serial passage in athymic nu/nu mice (NIH DCT/DTP Animal Production Program, Frederick, MD). Taxol resistance was previously induced *in vivo* via repeated treatment over several passage generations and maintained with periodic cycles of treatment. Individual mouse body weights for each experiment were within 2 g, and all mice were over 18 g at the start of therapy.

**Chemotherapy.** The animals were pooled and implanted bilaterally subcutaneously with 30–60 mg tumor fragments using a 12-gauge trocar and again pooled before unselective distribution to the various treatment and control groups. The tumors were allowed to grow to measureable size before the start of chemotherapy (day 7). Tumors were measured with a caliper two to three times weekly. Mice were sacrificed when the cumulative tumor burden reached 2000 mg. Tumor volumes were estimated from two-dimensional measurements [i.e., tumor mass (in mg) = (a x b^2^)/2, where “a” and “b” are the tumor length and width in mm, respectively]. For calculation of antitumor activity end points, both tumors on each mouse were added together, and the total mass per mouse was used.

**Antitumor activity analysis endpoints.** The following qualitative and quantitative end points were used to assess antitumor activities: (i) T/C and T-C (tumor growth delay) [where T is the median time in days required for the treatment group tumors to reach a predetermined size (e.g., 1000 mg) and C is the median time in days for the control group tumors to reach the same size; tumor-free survivors were excluded from the calculations]; and (ii) calculation of tumor cell kill [log_10_ cell kill total (gross) = (T—C)/(3.32)(Td), where (T—C) is the tumor growth delay, as described above, and Td is the tumor volume doubling time in days, estimated from the best fit straight line from a log-linear growth plot of control group tumors in exponential growth (100–800 mg range)]. Activity rating: For comparison of antitumor activity with standard agents and comparisons of activity between tumors, the log_10_ kill values were converted to an arbitrary activity rating [17]. For duration of treatment between 5–20 days: >2.8 log_10_ kill (Highly active ++++); 2.0–2.8 (+++); 1.3–1.9 (++); 0.7–1.2 (+); <0.7 (Inactive;–).

**Drug source, preparation and treatment details.** ABT-888 (dosed at 5mg/kg PO), CPT-11 (dosed at 45mg/kg IV) and Taxol (“Paclitaxel”, Hospira; dosed at 4.5–9mg/kg, IV) were prepared fresh for each injection according to package insert (all but Taxol diluted with 0.9% saline, USP as needed). Taxol was diluted from stock with water, USP. All treatment groups had 6 mice per group except the Diluent Control and ABT-888 single arm (n = 7 mice/group). The injection volumes were 0.2ml per IV injection or 0.1mL per PO injection (ABT-888) at the doses indicated. The treatment schedule was as follows: CPT-11: every 7 days for a total of 5 injections; ABT-888: twice a day for 2 cycles of 12 days; and Taxol: every two days repeated 10 times. Treatment began on day 7 (all but ABT-888 which started on day 9) post tumor implant (day 0).

**Statistical considerations for *in vivo* experiments.** The *in vivo* study design was a 2 x 2 factorial, with 2 main effects: CPT-11 (yes/no) and ABT-888 (yes/no). This yielded 4 treatment arms: CPT-11 alone, ABT-888 alone, the combination, or neither (i.e, control). Twenty-six mice (6 or 7 per treatment arm) underwent bilateral implantation with MX-1 tumor cells. This induced clustered or nested observations of subsequent tumor volume in the 52 mouse flanks. Tumor volume (in mm^3^) was measured on as many as 20 different days. Due to the modest number of mice (or mouse flanks), only a subset of 5 approximately equally spaced time points (days 6, 16, 27, 35, and 45 post-tumor implant) was used in the statistical longitudinal modeling. The treatment arms were imbalanced in size (vehicle control mice reached the maximum allowable tumor volume prior to the treated mice resulting in an incomplete data set for the 5 time points selected), and the primary sampling units (mouse flanks) were nested within mice. Together, these conditions required a nested incomplete repeated measures ANOVA using a mixed model approach. The two drugs were the fixed effects, and mouse flank was the random effect. Tumor volume at each of the 5 selected time points required a natural log (ln) transformation to achieve approximate Normality. However, for ease of interpretation, mean tumor size over time is presented (graphically) on the original scale of measurement (in mm^3^). The longitudinal modeling of tumor size was conducted using the MIXED procedure in SAS Version 9.3 [18].

Mean tumor volume was modeled as a function of 7 predictors: CPT-11 (yes/no), ABT-888 (yes/no), their interaction (a CPT-11*ABT-888 cross-product term); the linear effect of time (i.e., slope), and the interactions of time with CPT-11, ABT-888, and CPT-11*ABT-888. The last 3 terms yield tests of whether the model fitted slopes over time differ significantly by CPT-11 status (yes/no), or by ABT-888 status (yes/no), or by CPT-11 status and ABT status simultaneously.

The statistical modeling was done after preliminary analysis to find the best of 23 possible covariance structures for the 5 repeated measures of tumor volume based on smallest absolute value of Aikake’s Information Criterion (AIC). Comparisons and testing of differences in fitted slopes by treatment arms at each of the 5 time points were performed using ESTIMATE statements in the MIXED procedure in SAS. The resulting multiple comparisons issue was controlled for using the Holm procedure [19].

### Immunofluorescence

Fine needle aspiration biopsies were taken from MX-1 human tumor xenografts after 4 and 24 hours of CPT-11 (40mg/kg) and ABT-888 (5mg/kg) treatment. Each sample was washed twice and re-suspended with 400uL of PBS then centrifuged onto a glass slide and left to air dry for 15 min. Cells were fixed and permeabilized by immersing the slides into-20^o^C 1:1methanol/acetone (3 x 1 min immersion/1 min air dry). Slides were blocked overnight at 4^o^C with 5% bovine serum albumin in PBS and washed 3 times before incubation (2h) with anti γ-H2AX mouse monoclonal antibody (Millipore, MA, USA; 1:200 in 5% BSA in PBS). Slides were washed with PBS (3 x 5 min) before incubation with a goat anti-mouse FITC-conjugated secondary antibody (Sigma-Aldrich, MO, USA; 1:200, 1.5h). Following PBS washing (3 x 5 min), the slides were incubated with DAPI (Sigma-Aldrich, 2 mg ml^-1^; 5 min), washed in PBS (3 x 5 min), and mounted with Vectashield mounting media (Burlingame, CA, USA). The results were visualized and documented using the fluorescent setting of a Leica CTR5500 microscope (Leica Microsystems, IL, USA) and OpenLab software (Version 5; Improvision, IL, USA).

## Results

### *BRCA* mutated TNBC cell lines express high levels of PARP1 and are sensitive to PARP inhibition

**Fig 1:**
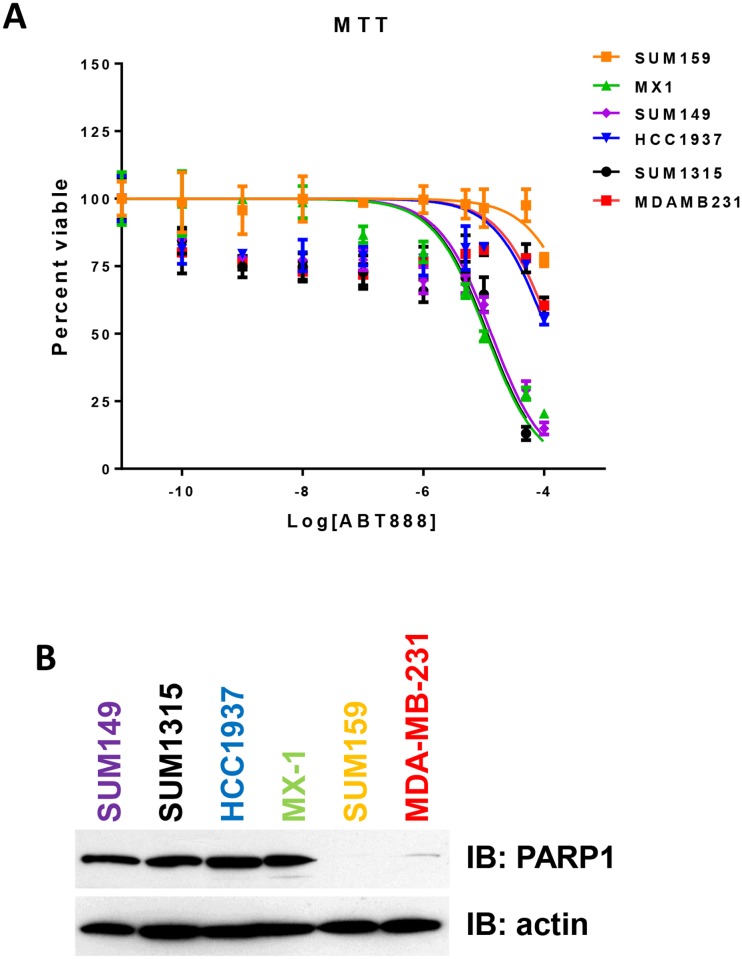
BRCA mutated TNBC cell lines express high levels of PARP1 and are sensitive to PARP inhibition. (A) Cells were treated with increasing concentrations of ABT-888 over a 5 day incubation period. MTT assays were used to assess cell viability. The fraction of surviving cells was used to calculate the IC_50_ values for ABT-888 for each cell line by sigmoidal dose response curve analyses (GraphPad Prism). IC_50_ values calculated from three independent experiments performed in triplicate were graphed for each cell line. (B) PARP1 protein expression levels were evaluated from cell lysates collected from cells growing in log phase. Equal protein was separated by SDS-PAGE, transferred to PVDF, and immunoblotted using anti-PARP1 antibodies. β-actin protein levels were used as a loading control.

Breast cancer cell lines and tumors containing *BRCA* mutations are as much as 1000 fold more sensitive to PARP inhibitors than wt-*BRCA* carriers [12,20–22]. Using six TNBC cell lines, four of which contained *BRCA* mutations, we found similar to published results using the PARP inhibitor ABT-888. Specifically, three of the four *BRCA* mutant cell lines had IC_50_ values for ABT-888 in the μM range for cell viability and in the nM range for clonogenic survival (Fig. 1A and S1A Fig.; Table 1). Again, as shown previously, the *BRCA* mutant cell lines have increased protein expression of PARP1 compared to the wt-*BRCA* cell lines (Fig. 1B). These results confirm the results of others demonstrating a trend for sensitivity to PARP inhibitors in tumor with *BRCA* mutants [12].

**Table 1:** TNBC cell lines with *BRCA* mutations respond to ABT-888 and CPT-11.

	MTT viability		Clonogenic survival				
	ABT888 IC_50_ (μM)	CPT-11 IC_50_ (μM)	ABT888 LD_50_ (μM)	CPT-11 LD_50_ (μM)	BRCA1 status	PARP levels	ERCC levels
**SUM149**	13.30	0.15	0.02	0.01	2288delT	++	-
**SUM1315**	9.01	0.13	0.09	0.13	185delAG	++	-
**HCC1937**	118.00	5.03	1.39	0.02	5382insC	++	++
**MX1**	10.90	8.02	0.93	0.15	3363delGAAA	++	++
**SUM159**	445.00	2.66	>100	0.86	wt	-	-
**MDAMB231**	138.00	19.90	32.97	0.27	wt	-	+

Cells were cultured in normal growth conditions in the presence of increasing concentrations of ABT-888 or CPT-11 for 72 hrs. MTT assays were used to determine the viable fraction of cells relative to the untreated controls. IC_50_ values were calculated using sigmoidal inhibitory response curves using GraphPad Prism. PARP and ERCC relative protein expression values:- = < 1, + = 1 to 3, ++ = > 3.

### Cell growth response of *BRCA* mutated TNBC cell lines to topoisomerase inhibition correlates with ERCC1 protein expression

**Fig 2:**
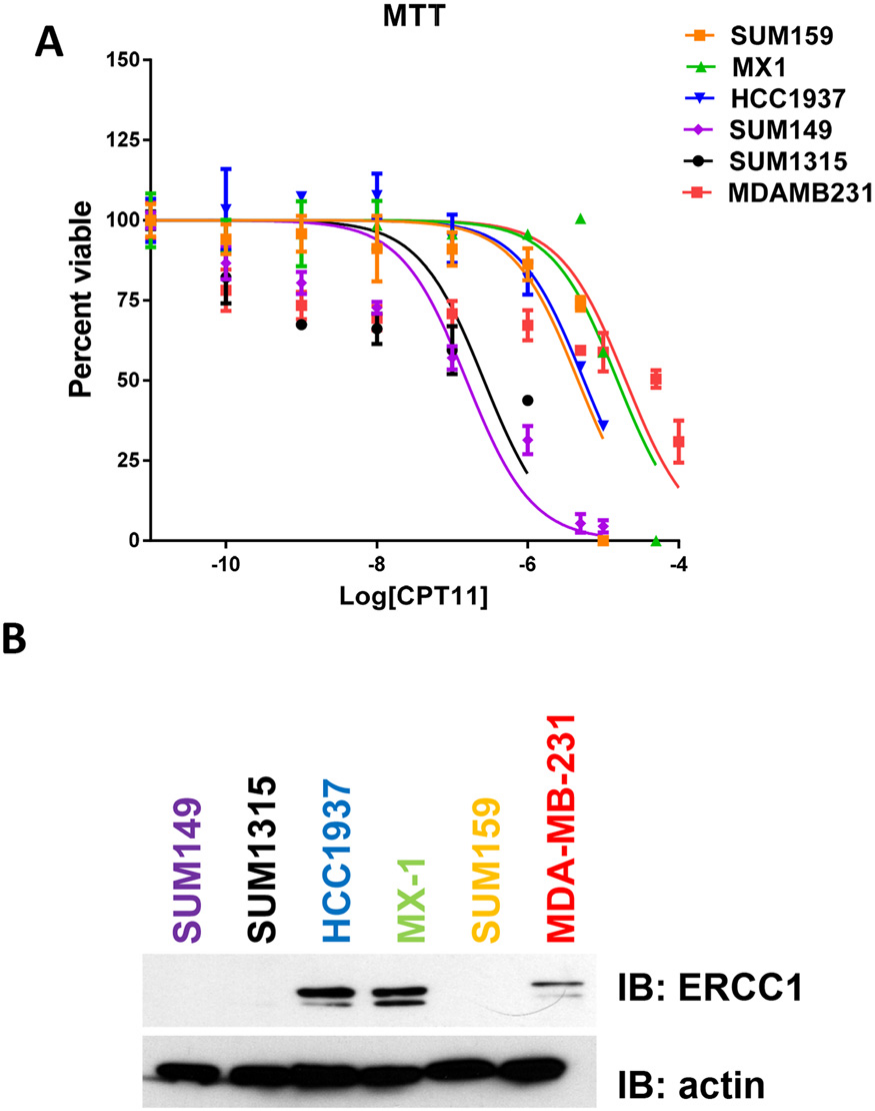
Response of BRCA mutated TNBC cell lines to topoisomerase inhibition correlates with ERCC1 protein expression. (A) Cells were treated with increasing concentrations of CPT-11 over a 5 day incubation period. MTT assays were used to assess cell viability. The fraction of surviving cells was used to calculate the IC_50_ values for ABT-888 for each cell line by sigmoidal dose response curve analyses (GraphPad Prism). IC_50_ values calculated from three independent experiments performed in triplicate were graphed for each cell line. (B) ERCC1 protein expression levels were evaluated from cell lysates collected from cells growing in log phase. Equal protein was separated by SDS-PAGE, transferred to PVDF, and immunoblotted using anti-ERCC1 antibodies. β-actin protein levels were used as a loading control.

**Fig 3:**
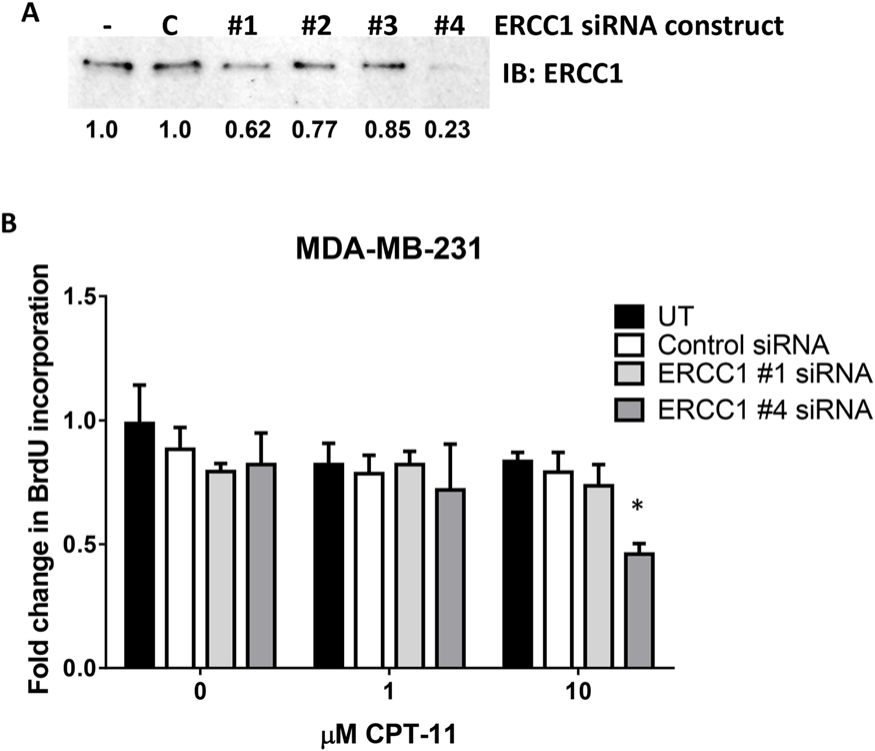
Knocking down ERCC1 protein expression sensitizes cells to CPT-11. (A and B) MDA-MB-231 cells were transfected with 4 non-overlapping siRNA oligos targeting ERCC1 as well as a negative control siRNA using Dharmafect. (A) Forty-eight hours later, cell lysates were prepared, SDS-PAGE separated the lysates, and immunoblotting was performed using anti-ERCC1 antibodies. (B) Cells were placed in serum free media for 18 hours and pulsed with BrdU to measure DNA synthesis. Cells were fixed, permeabilized with 2N HCl, neutralized with borate buffer, and blocked with 20% goat serum. BrdU incorporation was detected using anti-BrdU alexa fluor 624. BrdU positive cells were counted as a fraction of 100 cells counted/each of four fields/coverslip. Each experiment was performed in duplicate at least three times. * p-value = 0.022.

To determine if *BRCA* mutation status would predict response to DNA damage stimulated by inhibiting topoisomerase I with CPT-11, we performed cell viability assays to calculate the sensitivity of our panel of six TNBC cell lines to CPT-11. Two of the four TNBC cell lines with *BRCA* mutations as well as one of the *BRCA* wild-type cell lines, were sensitive at nM levels to CPT-11 when measuring cell viability and clonogenic survival (Fig. 2A and S1 Fig.; Table 1). The remaining three cell lines, including two *BRCA* mutant cell lines, had IC_50_ values for CPT-11 >100 μM when measuring cell viability (Fig. 2A and Table 1). Others have found that overexpression of nucleotide excision repair proteins, such as ERCC1 contributes to resistance to DNA damaging agents, specifically those that cause DNA cross-linking [23]. Therefore, we analyzed the protein expression levels of ERCC1. We found that ERCC1 protein expression corresponded with response to CPT-11, such that the higher the ERCC1 levels the higher the cell viability IC_50_ value for CPT-11 in the *BRCA* mutant cell lines (Fig. 2B). Importantly, knocking down ERCC1 protein expression in MDA-MB-231 cells increased the sensitivity of these cells to CPT-11, as measured by DNA synthesis (Fig. 3A and 3B). Taken together, these data demonstrate that TNBCs low levels of ERCC1 protein expression responded better to CPT-11.

### TNBC cell lines synergistically respond to the combination of PARP and topoisomerase inhibitors

To determine if there would be a beneficial effect combining these two drugs with efficacy in TNBC cell lines, we treated cells with varying doses of ABT-888 and CPT-11 in combination using the cell viability methods described above. Interestingly, using the Chou-Talalay method of determining synergy we found the HCC1937 and MX1 *BRCA* mutant cell lines were highly synergistic to the combination of CPT-11 and ABT-888 (Table 2; combinatorial index (CI) values < 1.0 are considered synergistic). These data suggest that the addition of CPT-11 to ABT-888 in these *BRCA* mutant TNBCs may increase biological response to treatment. In contrast however, SUM149 and SUM1315 *BRCA* mutant cell lines lacking ERCC1 protein expression had no added benefit to combining CPT-11 and ABT-888 (Table 2). The CI values for the SUM149 and SUM1315 cell lines suggest that the two drugs may actually have an antagonistic response. Taken together, these results suggest that ERCC1 expressing *BRCA* mutant TNBC cells lines have a synergistic response to the combination of ABT-888 and CPT-11.

**Table 2:** Summary ABT-888 and CPT-11 combination treatment in TNBC.

	CI Values MTT
**SUM149**	**3.28**
**SUM1315**	**3.93**
**HCC1937**	**0.13**
**MX1**	**0.44**
**SUM159**	**0.19**
**MDAMB231**	**0.11**

Combinatorial index values (CI) were determined using Calcusyn software from the cell viability data. CI values less than 1 indicate synergy. Each experiment was repeated three times in triplicate.

*Wt-BRCA* expressing TNBC cell lines did not respond to PARP inhibition (Fig. 1A). Therefore, we hypothesized that these cell lines would demonstrate an additive or synergistic response to the combination of topoisomerase I and PARP inhibitors. This hypothesis was based on the principle of synthetic lethality [8]. Again, using the Chou-Talalay method of determining synergy, we found that the *wt-BRCA* expressing TNBC cell lines were indeed highly synergistic to the combination of CPT-11 and ABT-888 as measured by cell viability assays and clonogenic assays (Table 2). Interestingly, this synergy was not as dependent of ERCC1 protein expression as observed with the *BRCA* mutant TNBC cell lines.

### The combination of PARP and topoisomerase inhibitors is an effective combination for TNBC *in vivo*

**Fig 4:**
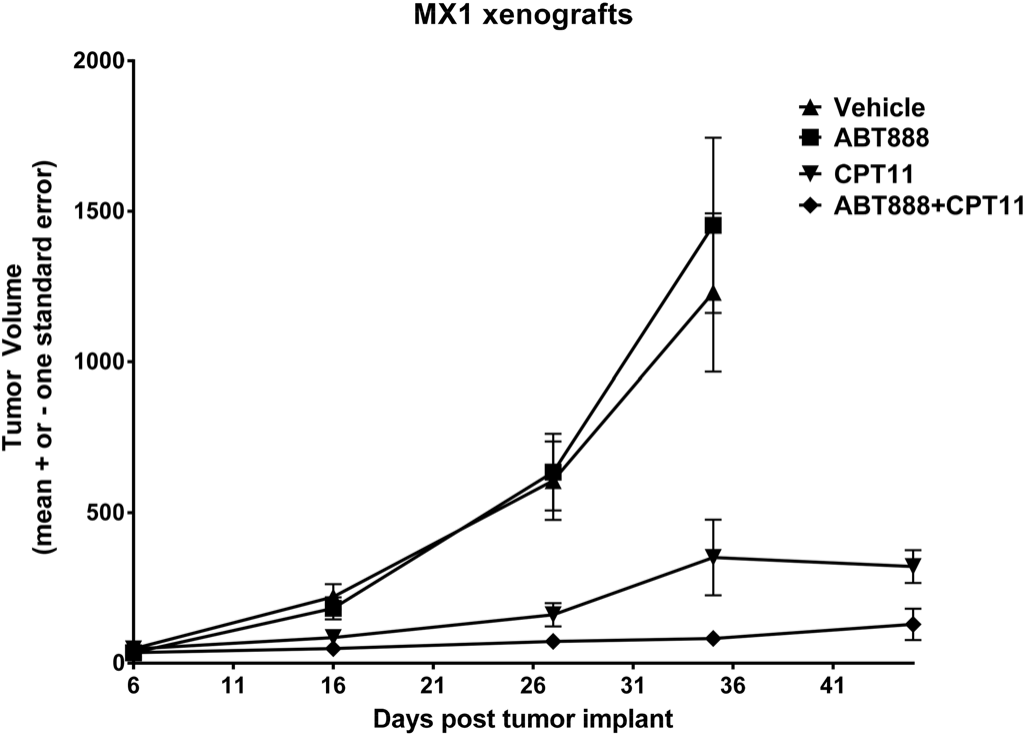
The combination of PARP and topoisomerase inhibitors is an effective combination for TNBC *in vivo*. MX-1 breast cancer xenografts were implanted between the front and hind leg of nude mice. Tumor volume (in mm^3^) was measured with calipers every two days, and body weight was taken bi-weekly. When tumors reached a measurable burden (~63 mm^3^), the indicated treatments were started. CPT-11 was given IV every 7 days in 5 doses for a total dosage of 225 mg/kg. ABT-888 was given PO twice a day from days 9 to 20 and again from days 23 to 28 for a total dosage of 240 mg/kg. Mean tumor volume (± standard error [SE] of the mean) is plotted over time, separately for each of the four drug treatment groups.

To validate these *in vitro* observations using a mouse model, *nude* mice were used to generate xenografts of the MX1 cells. These cells had ERCC1 protein expression in the context of a mutated *BRCA1* gene and *in vitro* were synergistically responsive to the CPT-11 and ABT-888 combination. Mice were treated with the indicated total dosage of drug for 31 days (Table 3). The toxicity of the drugs alone and in combination did not result in significant weight loss or death in the treatment cohorts (data not shown). The ABT-888 treated mice had similar tumor burden relative to the vehicle treated mice on Day 36 (the day after the last treatment day) and failed to show a tumor growth delay (Table 3). In contrast, CPT-11 alone reduced tumor burden relative to vehicle treated mice on Day 36 to 8% with a 30.5 day tumor growth delay (Table 3). Together these data show a significant response of MX-1 xenografts to CPT-11 with a gross log cell kill of 3.4 (Table 3). Importantly, in the presence of CPT-11, ABT-888 treatment was associated with a significant reduction in mean tumor size. The percent tumor volume at Day 36 was 0% of the control and the tumor growth delay was 40.5 days, indicating a highly effective drug combination (Table 3). Hence, as predicted from our *in vitro* data, the combination of PARP and topoisomerase inhibition provided the greatest decrease in tumor volume over time in the MX1 xenografts (Fig. 4). These data suggest that there might be efficacy of the ABT-888/CPT-11 combination in patients with TNBC.

**Table 3:** MX-1 xenografts respond to the combination of ABT-888 and CPT-11.

Treatment	Drug Route	Schedule	Total Dosage mg/kg	Drug death (day of death)	Median tumor burden in mg on d36 (range)	T/C%	Tumor free on d96	Time to 1000 mg in days (range)	Tumor growth delay (days)	Gross log cell kill	Notes
Diluent control	IV, PO	IV Q7x5 (start day 7) PO Bid d9, 20, 23–28 Qd37	-	-	2281 (775–5509)	-	0/7	27 (22–40)	-	-	-
CPT-11	IV	IV Q7x5 (start day 7)	225	0/6	190 (63–1206)	8	0/6	57.5 (55–62)	30.5	3.4	++++
ABT-888	PO	PO Bid d9, 20, 23–28 Qd37	240	0/7	2385 (895–4508)	105	0/7	28 (22–37)	1	< 0.5	-
Taxol	IV	Q2dx15(start day 7)	112.5	0/6	1867 (417–2620)	82	0/6	30.5 (26–57)	3.5	< 0.5	-
CPT-11+ ABT-888	IV, PO	IV Q7x5 (start day 7) PO Bid d9, 20, 23–28 Qd37	225, 240	0/6	0 (0–351)	0	4/6	67.5 (54–93)	40.5	4.5	++++

The indicated number of mice were treated by intravenous (IV) or oral (PO) administration of the indicated drug at the listed dosing schedule. Median tumor burden was determined at day 36 with the range of values present in parentheses. T/C% measures the growth of the tumors in the treated mice (T) compared to the diluent treated mice (C). The number of mice with unpalpable tumors are indicated by the tumor free on day 36 values. The tumor growth delay is calculated as the number of days in excess of the diluent treated mice required for the tumor to reach 1000 mg. Gross log cell kill is measured as (T—C)/(3.32)(Td) with T—C being the tumor growth delay and Td the calculated tumor doubling time (2.3 days). The pluses in the comments indicate biological efficacy of the drug treatment. Average Weight = 24.0 g/mouse.

### Measuring γH2AX phosphorylation as a biomarker for response to PARP and topoisomerase inhibitors

**Fig 5:**
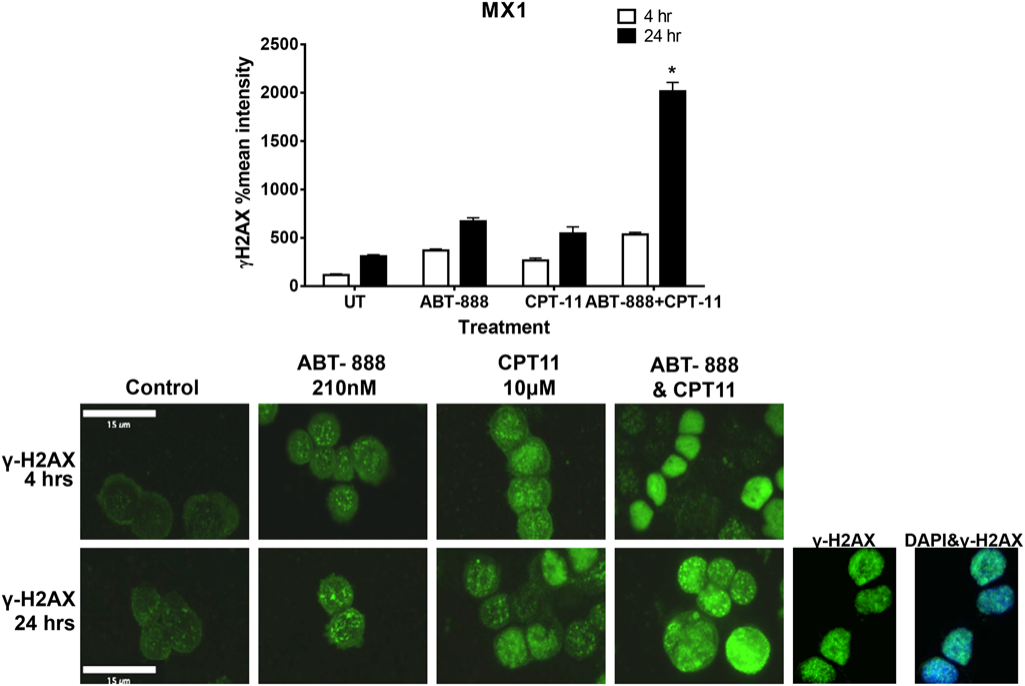
Measuring γH2AX phosphorylation as a biomarker for response to PARP and topoisomerase inhibitors. Biopsies from MX-1 human tumor xenografts after 4 and 24 hours of CPT-11 (40mg/kg) and/or ABT-888 (5mg/kg) treatment were taken and centrifuged onto a glass slide. Cells were fixed, permeabilized, blocked overnight, and incubated with anti γ-H2AX antibody. Slides were washed with PBS followed by staining with FITC-conjugated secondary antibody. Following PBS washing, the slides were incubated with DAPI, washed in PBS, and mounted. The results were visualized and documented using the fluorescent setting of a Leica CTR5500 microscope and quantified using OpenLab software. Each experiment was repeated three times representing the bars in the graph.

Markers of DNA damage are useful in determining the efficacy of DNA damaging agents both *in vitro* and *in vivo*. We measured γH2AX phosphorylation as a marker of double strand DNA breaks in the MX1 cell line after treatment with the drug combination. As predicted double strand DNA breaks were stimulated after 24 hr treatment with ABT-888 or CPT-11 alone (Fig. 5; 3-fold and 3.5-fold increase from untreated controls). Importantly, the combination of ABT-888 and CPT-11 increased γH2AX phosphorylation by 6.5-fold (Fig. 5; p-value < 0.0001). These data demonstrate that the synergistic decreases in cell viability observed in MX-1 cells with the combination of ABT-888 and CPT-11 is reflected by a significant accumulation of double strand DNA breaks.

## Discussion

Here we have found that TNBCs with *BRCA* mutations have enhanced sensitivity to PARP inhibitors, specifically in our experiments using ABT-888. Interestingly, we found that TNBCs with no detectable ERCC1 protein expression were particularly sensitive to CPT-11 when sensitivity was measured using a cell viability assay. These data were supported by knocking down ERCC1 protein expression and demonstrating an increase in sensitivity to CPT-11 as measured by a decrease in DNA synthesis. The combination of ABT-888 and CPT-11 resulted in synergistic decreases in cell viability in all TNBC cell lines tested, with the exception of the two cell lines most sensitive to CPT-11 as a single agent (Table 2). These data were strengthened by the demonstration that a TNBC cell line with a *BRCA* mutation showed decreased *in vivo* tumor growth with the combination of ABT-888 and CPT-11 in an animal model. Lastly, the phosphorylation of the DNA damage marker γ-H2AX was increased in MX-1 cells treated with both ABT-888 and CPT-11, compared to either drug alone. Taken together, these data provide support for the testing of ABT-888 and CPT-11 in TNBC patients.

ERCC1 is a DNA repair protein involved in the processes of nucleotide excision repair as well as homologous recombination. Specifically, ERCC1 binds to the 5’ end of damaged DNA, bringing with it XPF, an endonuclease [24]. The damaged DNA is then removed and replaced with the corrected base pairs. Previously, low expression of ERCC1 has been shown to correlate with resistance to platinum agents, including cisplatin [25]. Here we demonstrated that in the presence of *BRCA* mutations, low levels of ERCC1 protein expression correlated with a response to the topoisomerase I inhibitor, CPT-11. In contrast, high protein expression levels of ERCC1 in *BRCA* mutation carrying cell lines correlated with higher IC_50_ values for CPT-11. Interestingly, treating the ERCC1 expressing cells with ABT-888 or knocking down ERCC1 protein expression sensitized them to CPT-11. Similarly, Zhang and colleagues found in a colon cancer cell line, knocking down ERCC1 protein expression increased the response to cells to CPT-11 in combination with ABT-888 as measured by γ-H2AX phosphorylation [26]. These results are important for several reasons. First, these data demonstrate that the combination of ABT-888 and CPT-11 not only decreased cell viability and tumor growth, but did so by increasing double strand DNA breaks. In addition, these data suggest that measuring γH2AX phosphorylation from post-treatment patient biopsies may be an early predictor of therapeutic benefit.

It is estimated that 20% of TNBCs have *BRCA* mutations [27]. In our studies, we analyzed two *wt-BRCA* TNBC cell lines, SUM159 and MDA-MB-231, for response to PARP and topoisomerase inhibitors with the thought being that a similar synthetic lethality that occurs with PARP inhibitors and *BRCA* mutations may occur with ABT-888 in *wt-BRCA* cells treated with CPT-11. As expected, as a single agent, ABT-888 was not effective in TNBCs with *wt-BRCA* with calculated IC_50_ values in the high μM range. However, when comparing these two cell lines we found that lack of ERCC1 in SUM159 cells correlated with a log fold lower IC_50_ value for CPT-11. These data suggest that, together with the *BRCA mutant* cell line data, the protein expression of ERCC1 may be useful in predicting response to topoisomerase inhibitors in TNBCs. Interestingly, when ABT-888 and CPT-11 were added in combination, these *wt-BRCA* TNBC cells showed a high degree of synergy. Therefore, it can be surmised that loss of protein expression of ERCC1 in SUM159 may mimic some of the DNA damage repair defects seen with loss of BRCA protein function in *wt-BRCA* TNBC cells.

## Supporting Information

S1 Fig Clonogenic survival in response to ABT-888 or CPT-11. Cells were treated with increasing concentrations of ABT-888 or CPT-11 every other day. After 1 week, cells were trypsinized and replated at a low density in triplicate. Cells were cultured under normal growth conditions for 2 weeks. Colonies were stained using crystal violet and imaged using GelCount colony counter. Each experiment was repeated at least three times.
(TIF)
