# Supplementary figures and images for: Protein Expression of DNA Damage Repair Proteins Dictates Response to Topoisomerase and PARP Inhibitors in Triple-Negative Breast Cancer
Source: PLoS One. 2015 Mar 16;10(3):e0119614. doi: 10.1371/journal.pone.0119614 (PMC4361640; doi:10.1371/journal.pone.0119614)

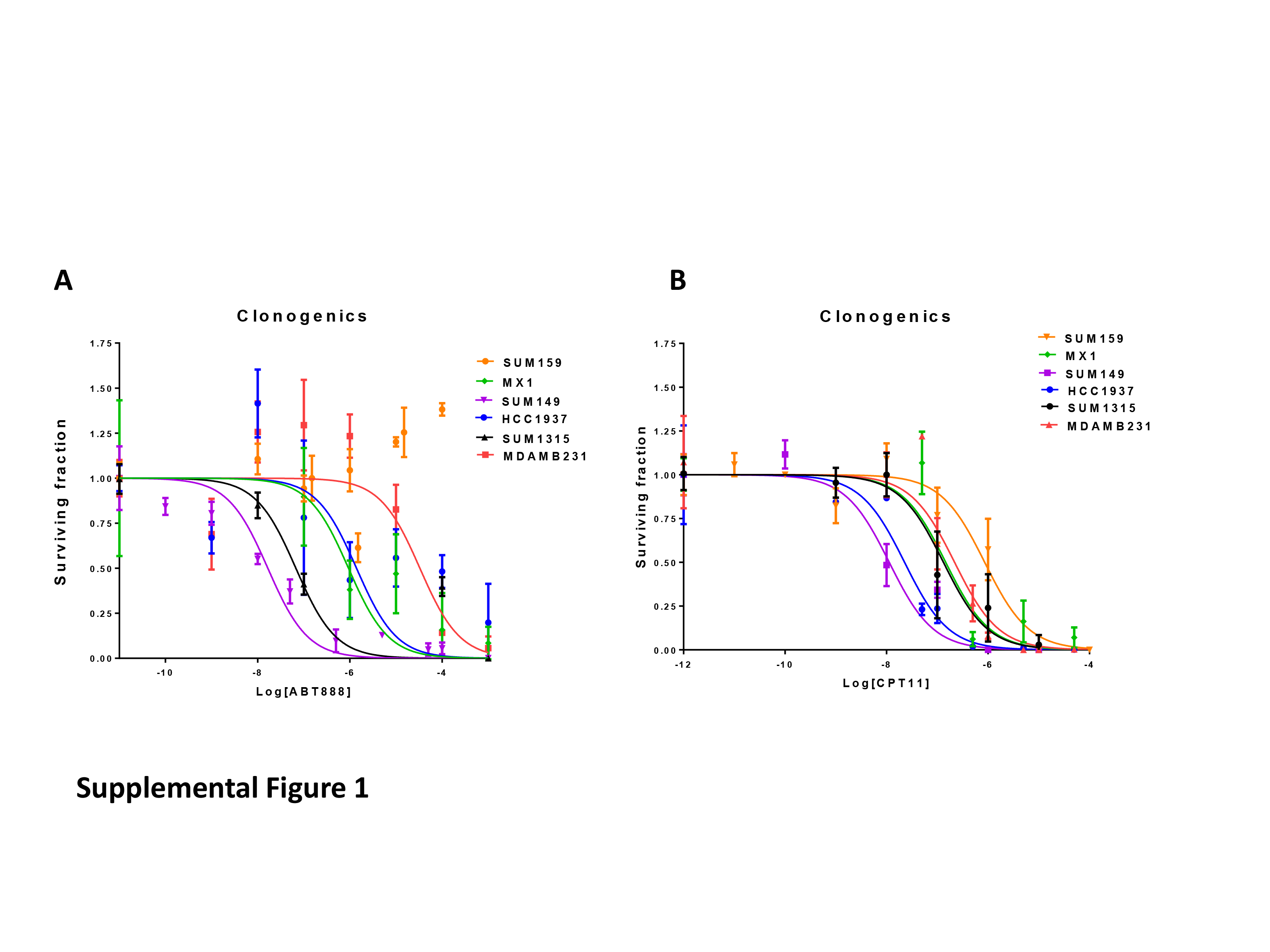

Supplement: S1 Fig — Cells were treated with increasing concentrations of ABT-888 or CPT-11 every other day. After 1 week, cells were trypsinized and replated at a low density in triplicate. Cells were cultured under normal growth conditions for 2 weeks. Colonies were stained using crystal violet and imaged using GelCount colony counter. Each experiment was repeated at least three times. (TIF) [file pone.0119614.s001.tif]
